# Prevalence of Poor Sleep Quality and Associated Factors Among Nurses in Southeast Iran: A Cross‐Sectional Study

**DOI:** 10.1002/hsr2.71542

**Published:** 2025-11-30

**Authors:** Raheleh Hashemi Habybabady, Hassan Okati‐Aliabad, Mahdi Mohammadi

**Affiliations:** ^1^ Health Promotion Research Center Zahedan University of Medical Sciences Zahedan Iran

**Keywords:** nurses, occupational stress, Pittsburgh Sleep Quality Index, sleep quality

## Abstract

**Background and Aims:**

Poor sleep quality is widespread among nurses, adversely affecting their well‐being and job performance, with various contributing factors. This study aimed to investigate the prevalence of poor sleep quality and its associated factors among nurses in Southeast Iran.

**Methods:**

A cross‐sectional study was conducted with 260 eligible nurses, randomly selected from hospitals affiliated with Zahedan University of Medical Sciences, using a multistage sampling method. Hospitals were stratified by type, wards grouped by specialty, and nurses randomly chosen from selected wards to ensure diverse institutional and work environment representation. Data were collected from April 20 to June 20, 2023. Sociodemographic information, lifestyle factors, work‐related characteristics, occupational stressors, and sleep quality were gathered. The Pittsburgh Sleep Quality Index (PSQI) was used to measure sleep quality, while the Occupational Role Questionnaire (ORQ) assessed occupational stressors. Simple and multiple logistic regression models were utilized to identify predictors of poor sleep quality.

**Results:**

Poor sleep quality was reported by 73.8% of nurses and was significantly associated with being married (OR = 1.82, 95% CI (1.02, 3.26); *p* = 0.04) and having over 15 years of work experience (OR = 9.81, 95% CI (2.06, 46.73); *p* = 0.004). Role overload (OR = 1.05, 95% CI (1.01, 1.11); *p* = 0.03) and role insufficiency (OR = 1.08, 95% CI (1.03, 1.13); *p* = 0.002) were significant predictors, while no associations were found with age, gender, education, BMI, exercise, working hours, having a second job, and shift work.

**Conclusion:**

The study underscores the high prevalence of poor sleep quality among nurses in Southeast Iran and identifies key contributing factors. These findings highlight the need for targeted interventions in nursing education and workplace policies to address sleep‐related issues, ultimately enhancing the well‐being of nurses and improving patient care outcomes.

## 1 Introduction

Sleep disturbance is a major concern among healthcare professionals, with prevalence rates significantly higher than those in the general population [1]. Nurses, as frontline caregivers, are particularly vulnerable to sleep disruptions due to the demanding nature of their work. The characteristics inherent to the nursing profession can negatively affect both sleep quality and overall quality of life [2]. Previous studies have shown that the working conditions of nurses can prolong sleep onset latency [3], while short rest periods between shifts often lead to fragmented sleep and, over time, contribute to shift work disorder and chronic fatigue [4].

Globally, poor sleep quality among nurses has been widely documented. A meta‐analysis reported that 61% of nurses experience poor sleep quality, with an average Pittsburgh Sleep Quality Index (PSQI) score of 7.13 ± 0.18 [5]. Similarly, studies in Jordan and Turkey found that 68% and 61.9% of nurses, respectively, had poor sleep quality [6, 7]. These findings highlight that sleep disturbance is a pervasive occupational health problem across healthcare systems.

Poor sleep quality among nurses leads to numerous negative consequences, including increased fatigue [8], burnout [9], and job dissatisfaction [10], while also reducing productivity [11], impairing cognitive and psychomotor performance [12, 13], and compromising patient safety through a higher risk of care and medication errors [14, 15, 16].

Multiple factors influence nurses' sleep quality, including occupational stress [17], work experience, working hours [18], shift work [19] and having a second job [20]. Furthermore, research indicates that nurses with longer durations of service are more likely to experience sleep disturbances [21]. In addition, demographic factors such as age [22], gender [21], and education [23] along with lifestyle factors like exercise [24] and BMI [25], also play significant roles in determining sleep quality.

Despite extensive global evidence, there is limited research examining sleep quality among nurses in underserved regions such as Southeast Iran, where healthcare resources and working conditions differ substantially from other contexts [26, 27]. Understanding these local dynamics is crucial for developing effective interventions. Therefore, the present study investigates the prevalence of poor sleep quality among nurses in Zahedan, Southeast Iran, and explores its association with demographic characteristics, lifestyle behaviors, work‐related factors (including shift work, working hours, second job, and job tenure), and occupational stress (specifically role overload and role insufficiency). The findings aim to inform targeted strategies and policies to enhance nurse well‐being and optimize patient care in resource‐limited settings.

## 2 Methods

### 2.1 Study Design and Participants

This cross‐sectional study investigated sleep quality and associated factors among nurses working in hospitals affiliated with Zahedan University of Medical Sciences in Southeast Iran. These hospitals serve as referral centers in Sistan & Baluchistan province, the second largest and most deprived region in Iran. Based on a pilot study estimating the prevalence of poor sleep quality at 79.6%, and using a type I error of 0.05 with an estimation error of 0.05, the required sample size was calculated to be 250. Ultimately, 260 nurses participated in the study.

Participants were selected randomly through a multi‐stage sampling method. In the first stage, affiliated hospitals were stratified by type (general or specialized), and a random sample was drawn from each stratum to ensure diverse institutional representation. In the second stage, wards within the selected hospitals were grouped according to specialty (e.g., internal medicine, surgery, intensive care), and a random selection of wards was made to reflect a range of nursing work environments. In the final stage, nurses working in the selected wards constituted the sampling unit. Lists of eligible nurses were obtained from ward rosters, and participants were randomly selected using a computer‐generated random number sequence to ensure fairness and equal probability of selection. Eligibility criteria included having more than 1 year of clinical work experience and providing informed consent. Nurses who were actively being treated for anxiety, depression, or sleep disorders with medication at the time of data collection were excluded from the study.

### 2.2 Data Collection

Data were collected from April 20 to June 20, 2023, using a self‐administered questionnaire, which included sections on sociodemographic characteristics, lifestyle factors, work‐related factors, occupational stressors, and sleep quality. Socio‐demographic characteristics included age, gender, marital status, and level of education (Diploma, Associate/BSc degree, and MSc degree). Two aspects of lifestyle, including BMI and exercise, were measured. Occupational characteristics, including years of work experience, daily working hours, second job, and work schedule (shift or non‐shift work), were collected.

The Persian version of the Pittsburgh Sleep Quality Index (PSQI) was utilized to evaluate overall sleep quality [28]. The PSQI is a widely used instrument designed to measure sleep quality and disturbances over 1 month. It is particularly useful in clinical and nonclinical settings for assessing sleep quality in various populations [29], including nursing staff [5]. The PSQI consists of 19 self‐rated items, which are grouped into seven components including subjective sleep quality (overall perception of their sleep quality, measured by one item), sleep latency (the time it takes for an individual to fall asleep, measured by two items), sleep duration (total amount of sleep obtained per night, measured by one item), habitual sleep efficiency (the ratio of total sleep time to time spent in bed, indicating how efficiently a person sleeps, measured by three items), sleep disturbances (frequency of sleep disruptions, such as waking up in the middle of the night, measured by 9 items), use of sleep medication (the frequency of using medication to help sleep, measured by one item), daytime dysfunction (the impact of sleep problems on daytime activities and functioning, measured by two items).

Components measured by a single item are scored directly from 0 to 3 or categorized into four levels with corresponding scores (0–3). For multi‐item components, scores are derived by summing or combining responses and then categorizing into four levels (0–3).

Each component is scored on a scale from 0 (no difficulty) to 3 (severe difficulty), and the scores for the seven components are then summed to yield a global PSQI score. The total score ranges from 0 to 21, with higher scores indicating worse sleep quality. A global score greater than 5 is commonly used to identify individuals with poor sleep quality [30].

Work stressors were measured using the Persian version of the Occupational Role Questionnaire (ORQ) [31]. The ORQ is a subsection of the Occupational Stress Inventory‐Revised Edition (OSI‐R) and comprises six scales designed to assess different dimensions of occupational stress including role overload (demands and responsibilities of job or role), role insufficiency (skills, knowledge, or abilities to meet the demands of the job), role ambiguity (understanding or specific information about job responsibilities, expectations, and performance criteria), role boundary (job responsibilities and the limits of role within an organization), responsibility (perceived level of responsibility in job roles), and physical environment (physical conditions and perception of these conditions as stressors) [32]. Since previous studies have shown that the “physical environment” component is not related to the hospital environment [33, 34], it was not considered in this study. Each scale consists of 10 items, scored on a 5‐point scale ranging from 1 (never) to 5 (very often). The scores for each scale were summed to provide a total score for that scale, with higher scores indicating greater levels of stress in that specific area. The overall ORQ score was obtained by summing the scores of the five scales. Thus, a higher ORQ score reflects greater overall work stressors.

### 2.3 Statistical Methods

Quantitative variables were described as mean ± standard deviation (SD), while qualitative variables were presented as frequency distributions. Simple and multiple logistic regression models, with odds ratios (ORs) and 95% confidence intervals (95% CIs), were used to identify predictors of poor sleep quality. The Forward LR method with a 0.05 entry probability was used in the multiple logistic model to select significant predictors. Poor sleep quality was defined as a PSQI score greater than 5. All statistical tests were two‐sided. A *p*‐value of less than 0.05 was considered statistically significant. All data analysis was performed using SPSS version 27.

### 2.4 Ethical Considerations

This study was conducted in accordance with the ethical standards outlined in the Helsinki Declaration. Ethical approval was obtained under the reference number IR.ZAUMS.REC.1400.261. All participants were informed about the objectives of the study and provided written consent before participation. Confidentiality and anonymity of the participants were maintained throughout the study. Participants were assured that their responses would be used solely for research purposes and that they could withdraw from the study at any time without any consequences. No identifying information was collected to ensure privacy and data protection.

## 3 Results

Most of the nurses were aged 20–35 years (70.8%), female (64.2%), and married (70%). Half of the participants had a normal weight, 72.4% had worked up to 10 years, and 85% held an undergraduate university degree. Most nurses reported working 6–8 h a day (82.3%) and sleeping up to 8 h a day (89.6%). Half of the nurses reported doing exercise, and 13.5% had a second job.

The mean scores of sleep quality and its components are presented in Table 1. Each component ranges from 0 to 3, with lower values indicating better sleep quality. Nurses reported relatively good sleep efficiency and low use of sleep medication. The mean scores of the remaining components ranged from 1.22 to 1.62, reflecting a moderate level of sleep quality.

**Table 1:** Frequency distribution and mean values of sleep quality and its subscales.

Variables	Categories	Scores	*N* (%)	Score values mean ± SD	Range of possible scores
Subjective sleep quality					
	Very good	0	31 (11.9)	1.47 ± 0.90	0–3
	Fairly good	1	116 (44.6)		
	Fairly bad	2	72 (27.7)		
	Very bad	3	41 (15.8)		
Sleep latency					
	Never	0	34 (13.1)	1.62 ± 0.89	0–3
	< 1 times a week	1	69 (26.5)		
	1–2 times a week	2	118 (45.4)		
	≥ 3 times a week	3	39 (15.0)		
Sleep duration (hours)					
	> 7 h	0	60 (23.1)	1.45 ± 0.98	0–3
	6–7 h	1	53 (20.4)		
	5–6 h	2	116 (44.6)		
	< 5 h	3	31 (11.9)		
Sleep efficiency					
	> 85%	0	180 (69.2)	0.57 ± 0.98	0–3
	75%–84%	1	36 (13.8)		
	65%–74%	2	19 (7.3)		
	< 65%	3	25 (9.6)		
Sleep disturbance					
	Never	0	16 (6.2)	1.37 ± 0.69	0–3
	< 1 times a week	1	147 (56.5)		
	1–2 times a week	2	82 (31.5)		
	≥ 3 times a week	3	15 (5.8)		
Use of sleep medication (per week)					
	Never	0	202 (77.7)	0.38 ± 0.78	0–3
	< 1 times a week	1	27 (10.3)		
	1–2 times a week	2	22 (8.5)		
	≥ 3 times a week	3	9 (3.5)		
Daytime dysfunction					
	Never	0	68 (26.2)	1.22 ± 0.96	0–3
	< 1 times a week	1	96 (36.9)		
	1–2 times a week	2	66 (25.4)		
	≥ 3 times a week	3	30 (11.5)		
Sleep quality					
	Good		68 (26.2%)	8.09 ± 3.61	0–21
	Poor		192 (73.8%)		

Abbreviation: SD, standard deviation.

Most of the nurses reported poor sleep quality (73.8%). According to the sleep quality subscales, 43.5% of nurses reported bad subjective sleep quality, and 86.9% experienced sleep latency. Sleep duration was 6 h or less in 56.5% of nurses. Additionally, sleep disturbances were reported by 93.8% of nurses, and 22.3% reported using sleep medication weekly (Table 1).

The odds of poor sleep quality were higher among married nurses compared to single ones (OR = 1.82, *p* = 0.04). However, no significant relationships were found between sleep quality and age, gender, or education (Table 2).

**Table 2:** Relationship between demographic factors and sleep quality.

Variables	Categories	Sleep quality		Simple logistic regression model	
		Good *N* (%)	Poor *N* (%)	OR (95% CI) for poor vs good sleep quality	*p* value
Age (year)					
	Equal to/less than 25	12 (30.8)	27 (69.2)	1.00	Ref.
	26–35	40 (27.6)	105 (72.4)	1.17 (0.54, 2.52)	0.70
	36–45	14 (25.0)	42 (75.0)	1.33 (0.54, 3.31)	0.54
	More than 45	2 (10.0)	18 (90.0)	4.00 (0.80, 20.04)	0.09
Gender					
	Male	30 (32.3)	63 (67.7)	1.00	Ref.
	Female	38 (22.8)	129 (77.2)	1.62 (0.92, 2.85)	0.10
Education					
	Diploma	11 (36.7)	19 (63.3)	1.00	Ref.
	Associate/BSc degree	55 (24.9)	166 (75.1)	1.75 (0.78, 3.90)	0.17
	MSc degree	2 (22.2)	7 (77.8)	2.03 (0.36, 11.52)	0.43
Marital status					
	Single	27 (34.6)	51 (65.4)	1.00	Ref.
	Married	41 (22.5)	141 (77.5)	1.82 (1.02, 3.26)	0.04

Abbreviations: CI, confidence interval; OR, odds ratio.

As BMI increased, there was a trend toward poorer sleep quality, though this relationship was not statistically significant. Additionally, there was no significant relationship between exercise and sleep quality (Table 3).

**Table 3:** Association between lifestyle factors and sleep quality.

Variables	Categories	Sleep quality		Simple logistic regression model	
		Good *N* (%)	Poor *N* (%)	OR (95% CI) for poor vs good sleep quality	*p* value
BMI					
	Thin	6 (37.5)	10 (62.5)	1.00	Ref.
	Normal	37 (27.8)	96 (72.2)	1.56 (0.53, 4.59)	0.42
	Overweight	23 (24.0)	73 (76.0)	1.90 (0.62, 5.81)	0.26
	Obese	2 (13.3)	13 (86.7)	3.90 (0.64, 23.60)	0.14
Exercise					
	No	30 (23.1)	100 (76.9)	1.00	Ref.
	Yes	38 (29.2)	92 (70.8)	0.73 (0.42, 1.27)	0.73

Abbreviations: CI, confidence interval; OR, odds ratio.

Poor sleep quality was more likely in nurses who had worked for more than 15 years compared to those with 1 year of job experience (OR = 9.81, *p* = 0.004). However, working hours, having a second job, and shift work were not significantly related to sleep quality (Table 4).

**Table 4:** Association between work‐related factors and sleep quality.

Variables	Categories	Sleep quality		Simple logistic regression model	
		Good *N* (%)	Poor *N* (%)	OR (95% CI) for poor vs good sleep quality	*p* value
Work experience (years)					
	1	15 (36.6)	26 (63.4)	1.00	Ref.
	2–5	25 (29.1)	61 (70.9)	1.41 (0.64, 3.10)	0.40
	6–10	14 (23.0)	47 (77.0)	1.94 (0.81, 4.63)	0.14
	11–15	12 (33.3)	24 (66.7)	1.15 (0.45, 2.95)	0.76
	More than 15	2 (5.6)	34 (94.4)	9.81 (2.06, 46.73)	0.004
Working time (hours)					
	6–8	60 (28.0)	154 (72.0)	1.00	Ref.
	More than 8	8 (17.4)	38 (82.6)	1.85 (0.82, 4.20)	0.14
Second job					
	No	58 (25.8)	167 (74.2)	1.00	Ref.
	Yes	10 (28.6)	25 (71.4)	0.87 (0.39, 1.92)	0.73
Shift work					
	No	7 (17.9)	32 (82.1)	1.00	Ref.
	Yes	61 (27.6)	160 (72.4)	0.57 (0.24, 1.37)	0.21

Abbreviations: CI, confidence interval; OR, odds ratio.

The odds of poor sleep quality increased with higher role overload (OR = 1.07, *p* = 0.006), greater role insufficiency (OR = 1.09, *p* = 0.001), and higher levels of total occupational stress (OR = 1.02, *p* = 0.008). According to the multiple model, poor sleep quality was more likely in nurses experiencing increased role overload (OR = 1.05, *p* = 0.03) and greater role insufficiency (OR = 1.08, *p* = 0.002). These occupational stress components remained significant predictors of poor sleep quality even after adjusting for marital status and work experience (Table 5).

**Table 5:** Association between occupational stress and sleep quality.

Variables	Sleep quality		Simple logistic regression model^a^		Multiple logistic regression model^b^		Adjusted multiple logistic regression model^c^	
	Good	Poor	OR (95% CI)	*p* value	OR (95% CI)	*p* value	OR (95% CI)	*p* value
Role overload	27.75 ± 5.75	30.22 ± 6.33	1.07 (1.02, 1.12)	0.006	1.05 (1.01, 1.11)	0.03	1.06 (1.01, 1.11)	0.02
Role insufficiency	26.62 ± 6.66	29.69 ± 5.75	1.09 (1.04, 1.14)	0.001	1.08 (1.03, 1.13)	0.002	1.09 (1.03, 1.14)	0.001
Role ambiguity	26.88 ± 7.13	26.88 ± 6.14	1.00 (0.96, 1.05)	> 0.99				
Role boundary	26.97 ± 5.02	27.45 ± 5.63	1.02 (0.97, 1.07)	0.54				
Responsibility	28.18 ± 5.07	28.93 ± 5.33	1.03 (0.97, 1.08)	0.31				
Total occupational stress	136.40 ± 17.02	143.18 ± 17.86	1.02 (1.006, 1.04)	0.008				

Abbreviations: CI, confidence interval; OR, odds ratio.

a Simple logistic model with one independent variable.

b Multiple logistic model excluding total occupational stress.

c Multiple logistic regression model including two independent variables—role overload and role insufficiency—adjusted for marital status and work experience.

## 4 Discussion

This study investigated the prevalence and determinants of poor sleep quality among nurses in Zahedan, Southeast Iran. The findings revealed that 73.8% of nurses experienced poor sleep quality, with a mean PSQI score of 8.09, indicating a substantial burden of sleep‐related problems. This prevalence aligns with studies from Jordan (68%) [6], Ethiopia (75.5%) [35], and China (55%) [36], demonstrating that poor sleep quality is a widespread issue among nurses worldwide. However, the slightly higher prevalence in this study may reflect the socioeconomic challenges, limited healthcare resources, and heavy workloads that characterize healthcare systems in low‐resource settings like Southeast Iran.

The PSQI component scores further highlight specific areas of concern, including sleep latency, duration, and disturbances. The mean scores for subjective sleep quality (1.47), sleep latency (1.62), and sleep duration (1.45) indicate moderate to poor sleep outcomes, consistent with international findings [5].

The current study found that 44.6% of nurses reported fairly or very bad subjective sleep quality, 86.9% experienced prolonged sleep latency, and 11.9% slept less than 5 h per night. Additionally, 93.8% of nurses experienced sleep disturbances, and 22.3% reported using sleep medication weekly. These findings suggest that occupational demands have a substantial negative impact on nurses' sleep health. These findings are similar to reports from Ethiopia, where 89.6% had sleep latency issues and 9.4% slept less than 5 h per night, 91% experienced sleep disturbances, and 14.7% reported using sleep medication weekly [35]. Such cross‐country similarities suggest that systemic workplace stressors are common drivers of poor sleep among nurses globally, particularly in low‐ and middle‐income countries (LMICs).

Marital status was significantly associated with poor sleep quality, with married nurses reporting poorer sleep. This may be due to additional familial and caregiving responsibilities, particularly among women [37]. Consistent with prior studies from Jordan and Iran, no significant associations were found between sleep quality and age, gender, or education level [18, 38], suggesting that occupational stressors and family demands may play a more influential role than demographic factors.

In this study, nurses with longer job tenure (over 15 years) also showed a higher likelihood of poor sleep quality, corroborating findings from other studies [21]. Longer job tenure may be associated with increased job strain and burnout among nurses, both of which can negatively affect sleep duration [39]. Conversely, this study did not find a significant relationship between working hours, having a second job, and shift work with poor sleep quality. This result aligns with some previous studies [18, 20, 40]. However, McDowall et al. (2017) found that the prevalence of poor sleep quality was significantly higher in shift‐working nurses (78%) compared to non‐shift‐working nurses (59%), with shift work being the only significant factor associated with poor sleep quality after controlling for the other variables [41]. This discrepancy may be attributed to differences in the study populations, which can affect the relationship between shift work and sleep quality. In the present study, 13.80% of participants had more than 15 years of nursing experience, whereas in the study by McDowall et al., 51.76% of nurses had over 15 years of experience.

Although an upward trend between BMI and poor sleep quality was observed, the relationship was not statistically significant. Similar results have been reported in India [42] that found that underweight, overweight, and obese nurses had poorer sleep quality compared to their counterparts with normal BMI. Despite the observed trend, the association was not statistically significant, mirroring the results of our study.

The study also found no significant relationship between exercise and sleep quality. While physical activity is widely known to promote better sleep outcomes [43, 44], the absence of a significant association here may be attributed to variations in exercise type, duration, and timing. Given the demanding schedules of nurses, irregular or insufficient physical activity may limit its potential benefits on sleep.

In this study, occupational stress, specifically role overload and role insufficiency, was a strong predictor of poor sleep quality. Each one‐unit increase in these stress components increased the odds of poor sleep by 5% and 8%, respectively. These findings are consistent with previous studies demonstrating that occupational stress is one of the most critical determinants of sleep problems among healthcare workers [45, 46, 47]. In Southeast Iran, where hospitals are often understaffed and nurses face high workloads with limited institutional support, stress‐induced sleep disturbances are likely exacerbated.

### 4.1 Practical Implications

The findings of this study underscore the urgent need for organizational and educational interventions to improve nurses' sleep health. Implementing stress management training programs could help nurses better cope with occupational demands. Shift scheduling adjustments may also mitigate sleep deprivation and fatigue. Additionally, sleep hygiene education programs should be incorporated into nursing curricula and workplace health initiatives to promote behaviors conducive to better sleep.

### 4.2 Limitations

This study has several limitations that should be considered when interpreting the findings. First, due to its cross‐sectional design, the study cannot establish causal relationships between poor sleep quality and the identified predictors. The observed associations reflect correlations at a single point in time, making it unclear whether the contributing factors, such as shift work or occupational stress, are causes or consequences of poor sleep quality.

Second, the study relied entirely on self‐reported data for key variables, including sleep quality, work hours, and occupational stressors. This introduces the possibility of recall bias, as participants may not accurately remember or report past experiences or behaviors. Additionally, social desirability bias may have influenced some responses, particularly in relation to work performance or sleep habits, leading to potential underreporting or overreporting of certain conditions. These biases may affect the reliability of the data and the strength of the reported associations.

Third, the study was conducted in a specific geographic area—hospitals affiliated with a single university in Southeast Iran—which may limit the generalizability of the results to other regions or healthcare systems with different organizational structures, work cultures, or staffing patterns.

Finally, although the study utilized validated instruments such as the Pittsburgh Sleep Quality Index (PSQI) and the Osipow Occupational Stress Questionnaire (ORQ), these are subjective measures and may not fully capture the complexity of sleep disturbances or occupational stress. Objective assessments, such as actigraphy or physiological stress markers, could provide more accurate data.

Future studies employing longitudinal designs, objective measurement tools, and broader, more diverse populations are recommended to validate and expand upon these findings.

## 5 Conclusion

This study revealed a high prevalence of poor sleep quality among nurses, significantly influenced by occupational stressors such as role overload and role insufficiency, as well as demographic factors like marital status and job tenure. Targeted interventions, including stress management training, optimized shift scheduling, and sleep hygiene education, are essential to improve nurses' sleep health, enhance their well‐being, and promote safer, higher‐quality patient care.

## Author Contributions

**Raheleh Hashemi Habybabady:** conceptualization, project administration, resources, data curation, writing – review and editing. **Hassan Okati‐Aliabad:** conceptualization, methodology, validation, data curation, writing – original Draft. **Mahdi Mohammadi:** conceptualization, supervision, formal analysis, writing – review and editing. All authors have read and approved the final version of the manuscript. The corresponding author had full access to all of the data in this study and takes complete responsibility for the integrity of the data and the accuracy of the data analysis.

## Disclosure

The lead author Hassan Okati‐Aliabad affirms that this manuscript is an honest, accurate, and transparent account of the study being reported; that no important aspects of the study have been omitted; and that any discrepancies from the study as planned (and, if relevant, registered) have been explained.

## Conflicts of Interest

The authors declare that they have no known competing financial interests or personal relationships that could have appeared to influence the work reported in this paper.

## Data Availability

The data that support the findings of this study are available from the corresponding author upon reasonable request.
